# Community‐based Acceptance and Commitment Therapy Programmes for rheumatic conditions: An acceptability and qualitative process evaluation study

**DOI:** 10.1111/bjhp.70031

**Published:** 2025-10-23

**Authors:** Vasiliki Christodoulou, Katerina Artemiou, Vasilis S. Vasiliou

**Affiliations:** ^1^ University of Central Lancashire, Cyprus Pyla Cyprus; ^2^ Clinical Psychologist, Private Practice Nicosia Cyprus; ^3^ Department of Psychology, Royal Holloway University of London London UK; ^4^ Nuffield Department of Orthopaedics, Rheumatology and Musculoskeletal Sciences University of Oxford Oxford UK

**Keywords:** acceptability, ACT group, brief ACT intervention, chronic health conditions, process evaluation, programme length, qualitative, rheumatisms

## Abstract

**Purpose:**

Dissemination and implementation of socially prescribed community‐based programmes for individuals with rheumatic conditions remain rare. However, such programmes can help overcome key barriers, including limited access to evidence‐based psychological interventions, individuals' preference for psychosocial care outside of rheumatology clinics and the prevention of isolation and loneliness.

**Methods:**

This study presents a qualitative process evaluation of a community‐based Acceptance and Commitment Therapy (ACT) group intervention, delivered as part of a psychosocial service within a support organization for individuals with rheumatic conditions. We conducted 12 semi‐structured qualitative interviews following participants' completion of five in‐person ACT group sessions. Reflexive thematic analysis was used to assess acceptability and explore how participants conceptualized ACT's processes of change.

**Results:**

Four key themes emerged, offering practical considerations for planning and delivering ACT groups for individuals with rheumatic conditions: (1) the process of finding peace through mindfulness while managing practice‐related difficulties, (2) recognizing the importance of making values‐consistent choices, (3) navigating an ambivalent relationship with pain and (4) the dual nature of the group experience—both comforting and awkward.

**Conclusions:**

Findings highlight the implications of programme duration in planning ACT groups for individuals with rheumatic conditions in the community. The study suggests that acceptance and mindfulness may be time‐bound and context‐sensitive processes, influenced by the fluctuating symptomatology of rheumatic conditions. Mindfulness is best developed step by step, starting with body awareness, understanding symptoms and slowly bringing mindfulness into daily life. Pain acceptance should focus more on facilitating momentary patterns of activity engagement, rather than willingness towards the fluctuating symptoms.

**Statement of Contribution**

**What is already known on the subject?**

- Psychological coping processes can enhance functioning in individuals with rheumatic condition.
- Acceptance and commitment therapy (ACT) demonstrates improvements in pain, fatigue and disability.
- Socially prescribed and community‐based self‐care programmes can support when formal services are unavailable.

**What does this study add?**

- Cultivating body awareness and adaptive pain meanings is essential before introducing mindfulness in rheumatic care.
- Mindfulness and pain acceptance follow a dynamic and cyclical trajectory in chronic rheumatic pain.
- Values clarification enables renegotiation with neglected commitments under the limitations of rheumatic conditions.
- Community‐based support offers a feasible, acceptable step‐care psychosocial service.

## INTRODUCTION

Rheumatic conditions, including osteoarthritis and rheumatoid arthritis, affect over 24% of the global population (Cross et al., 2014). These chronic conditions cause physical and psychosocial problems for individuals and their families (Talarico et al., 2023), leading to diminished quality of life and impaired daily functioning (Cai et al., 2023). Fortunately, the advent of the new modified monoclonal antibody biologic agents, such as TNF inhibitors, has improved the management of key rheumatic body‐focused symptoms, including joint tenderness, stiffness, swelling and functional impairment (Buckley et al., 2020; Lwin et al., 2020; Senolt, 2019). However, the nature of the rheumatic conditions is characterized by immune‐driven joint inflammation, fluctuating symptoms and ongoing side effects from immunosuppressant glucocorticoid treatments (Theocharous, 2025) that disrupt the physiological stress response system (Evers et al., 2014) and the central nervous system regulation (Gao et al., 2022). This results in many individuals experiencing relapse‐remitting symptoms, leading to reduced physical functioning (Walsh & McWilliams, 2014), hindered treatment progress (Dures et al., 2016) and worsened mental health (Lwin et al., 2020; Ziarko et al., 2019). Ongoing research indicates that factors beyond disease activity and pharmacological treatment—such as interpersonal and contextual influences—play a crucial role in improving disease management (Santiago et al., 2015). It is with this in mind that the UK National Institute for Health and Care Excellence (NICE) for rheumatic disease management recommends addressing these broader factors to optimize treatment outcomes (NICE, NG100, 2018).

Amounting evidence indicates that targeting key psychological processes—particularly shifting illness perceptions to view symptoms as less catastrophic and adopting effective coping processes—can enhance functioning in individuals with rheumatic conditions (Marks, 2014; van Middendorp & Evers, 2016). However, a recent meta‐analysis indicates that coping alone may not alleviate daily challenges (Hinch & Sirois, 2024). Fostering resilience and flexibility through higher order transdiagnostic psychological processes, such as psychological flexibility (Hardy & Segerstrom, 2017; McCracken et al., 2012; Trainor et al., 2019), mindfulness (Zhou et al., 2020) and self‐compassion (Kılıç et al., 2021) can help individuals better manage the daily rheumatic symptom fluctuations.

Interventions targeting these processes have been tested in randomized controlled trials demonstrating their effectiveness (Nagy et al., 2023). Cognitive Behavioural Therapy (CBT) remains the most extensively researched and evidence‐based approach (Shen et al., 2020), along with contextual behavioural interventions, such as mindfulness‐based practices (DiRenzo et al., 2018), and acceptance and commitment therapy (ACT) (Hegarty et al., 2020). These interventions have demonstrated improvements in pain, fatigue, and disability by fostering self‐management and flexible perspectives on the condition (Bekarissova et al., 2024). Regarding ACT specifically, a systematic review (Hegarty et al., 2020) identified 10 RCTs evaluating its effectiveness in individuals either diagnosed with a rheumatic disease (five studies: Clarke et al., 2017; Luciano et al., 2014; Simister et al., 2018; Steiner et al., 2013; Wicksell et al., 2013) or living with chronic pain, of which 10%–30% had chronic pain attributed to rheumatism (Hegarty et al., 2020). Across these trials, ACT—most often delivered in group format—was compared with treatment as usual, active comparators, or passive controls. Results showed consistent improvements in favour of the ACT versus controlled groups in outcomes such as disability, anxiety, and depression, with many effects maintained up to 6 months, alongside reductions in pain intensity. Similar outcomes were observed in the subset of studies focusing specifically on rheumatic conditions (Clarke et al., 2017; Luciano et al., 2014; Simister et al., 2018; Steiner et al., 2013; Wicksell et al., 2013). Notably, two RCTs that included active control conditions reported significantly greater improvements in disability for the ACT condition, both immediately post‐treatment and at 6‐month follow‐up. Regarding intervention delivery, the included ACT intervention studies in the review varied in length, with a minimum of four sessions. The total duration ranged from 8 to 20 h. Overall, these findings corroborate with previous systematic reviews, such as Graham et al. (2016) Konstantinou et al. (2023) and Carvalho et al. (2024), suggesting that ACT can improve quality of life in individuals with rheumatic diseases, though barriers to broader implementation—as noted below—largely remain.

Firstly, most interventions have been tested in research contexts, with limited applications in the community where most individuals with rheumatic conditions receive care (Dures et al., 2016). Secondly, access is often limited by a lack of professionals trained in the psychosocial care of rheumatic conditions (Hale & Treharne, 2010). Thirdly, some individuals may find formal interventions impractical, preferring alternatives to conventional consultations with mental health professionals (Sturgeon et al., 2016). Given these challenges, scholars emphasize the need to integrate community‐based psychosocial interventions into rheumatology care (Bekarissova et al., 2024; Bokhari & Mushtaq, 2023; Dures et al., 2016). Socially prescribed and community‐based self‐care programmes may better accommodate diverse needs (Karp et al., 2023) by providing practical psychosocial support for individuals with rheumatic conditions, especially where formal services are unavailable. So far, there is limited research on community‐based interventions designed for rheumatic conditions.

Existing evidence from other contexts suggests that combining psychoeducational and experiential workshops with brief and flexible delivery formats can lead to positive outcomes in targeted health behaviours, such as functioning (Ahn et al., 2013; Christodoulou et al., 2021, 2024; Ory et al., 2013). For example, a meta‐analysis from Dochat et al. (2021), examining single‐session behavioural interventions for chronic health conditions, reported medium‐to‐large effects on functioning and mental health outcomes. Similar benefits have been observed in other agile and community‐based interventions for chronic pain (Taylor et al., 2016), skin conditions (Powell et al., 2023) and cancer adjustment (Keenan et al., 2024). However, research addressing the unique challenges of rheumatic conditions in such an accessible format remains limited.

The need for accessible, out‐of‐clinic psychosocial interventions underscores the importance of advancing community‐based programmes through implementation research that improves delivery, outcomes and engagement among individuals not accessing standard care (Ammerlaan et al., 2017). Such programmes should target self‐management of the fluctuating symptoms of rheumatic conditions, rather than solely targeting functional outcomes (Dougados et al., 2015). Acceptance and commitment therapy (ACT) is a psychological approach that is considered transdiagnostic, flexible in delivery (Dindo et al., 2017), and process‐based. ACT fosters evidence‐based coping and self‐care strategies (McCracken, 2023) and has a plethora of empirical evidence for managing chronic physical conditions (Konstantinou et al., 2023).

ACT cultivates psychological flexibility—the ability to be open, aware and behaviourally adaptive in response to lived experiences while pursuing personally meaningful life directions (Hayes et al., 2011; McCracken, 2024). For instance, people with rheumatic conditions often experience physical strains (e.g., joint pain) and bothering thoughts (e.g., ‘walking is too hard’). Responding with psychological inflexibility can lead to isolation and withdrawal from valued activities, whereas a psychologically flexible response fosters engagement in meaningful activities with an awareness of the rheumatic‐related limitations.

To promote flexibility and enhance daily functioning, ACT combines acceptance and mindfulness processes with behaviour‐change techniques. Evidence supports ACT's efficacy across various chronic conditions, exhibiting fluctuating symptomatology (Graham et al., 2016), including chronic pain (Martinez‐Calderon et al., 2024), headaches (Vasiliou et al., 2021, 2022), skin conditions (Powell et al., 2023) and diabetes (Sakamoto et al., 2022), among others. In the context of rheumatic conditions, ACT has shown benefits for individuals with rheumatic arthritis (Maher‐Edwards et al., 2020), osteoarthritis (Clarke et al., 2017) and fibromyalgia (Luciano et al., 2014; Simister et al., 2018; Steiner et al., 2013; Wicksell et al., 2013), with studies demonstrating sustained improvements in disability, distress and quality of life for up to 6 months compared to control groups (Hegarty et al., 2020).

Despite promising findings, there is an urgent need for more context‐specific research on ACT's feasibility and acceptability for rheumatic conditions (Hegarty et al., 2020), because these programs are frequently employed as community‐based supporting approaches, outside clinical settings (Simpson et al., 2023). To date, no qualitative studies have explored participants' experiences in ACT groups specifically for rheumatic conditions. However, evidence from studies of ACT in other chronic conditions—including chronic pain (Bendelin et al., 2020; Thompson et al., 2018), cancer (Keenan et al., 2024; Köhle et al., 2017), and neurological conditions (Storey et al., 2025)—suggests that participants commonly describe ACT‐consistent processes in their narratives, such as greater psychological flexibility, prioritizing self‐care, values‐based decision making, and shifts in attitudes towards symptoms. Understanding how such processes are reflected in ACT groups for rheumatic conditions may provide valuable insight into mechanisms of change that are not easily captured in quantitative studies (Lewin et al., 2009).

The present study examined how individuals living with a rheumatic condition engaged with a brief ACT group intervention when packaged, delivered, and disseminated as a community‐based approach, offered by a charitable organization. The study had two primary aims: (1) to assess the acceptability of the intervention, including participants' willingness to engage with a community‐based programme; and (2) to identify how participants formed explanations about ACT's processes of change.

## METHODS

### Research design

Following recommendations for evaluating health‐related interventions (Moore et al., 2015; Skivington et al., 2021), we conducted a qualitative process evaluation of acceptability using a ‘person‐based approach’ (Yardley et al., 2015). The study did not include a comparison control group or analysis of quantitative data and was not part of a nested RCT. It entirely focused on process qualitative interview data. We examined participants' perceptions of the intervention's delivery suitability to their physical health and group interactions. To assess acceptability, we employed the Theoretical Framework of Acceptability (TFA), which includes affective attitude, burden, perceived effectiveness, ethicality, intervention coherence, opportunity costs and self‐efficacy (Sekhon et al., 2017). We also explored how participants perceived the ACT components in supporting adaptation, following a process evaluation (Moore et al., 2015).

Our epistemological stance was rooted in social constructivism (Braun & Clarke, 2022a; Gergen, 2015; Joffe, 2012) with the aim to identify how participants constructed their understanding of the intervention. This was supported by a social constructivist theoretical framework, emphasizing that learning and meaning making are socially situated processes (Shepard, 2000). The study received ethical approval from the Cyprus Bioethics Committee (EEBKEΠ 2021.01.201). We employed the Standards for the Reporting of Qualitative Research Guidelines (SRQR; O'Brien et al., 2014).

### Procedures

Participants were recruited via flyer and email advertisements, distributed by the rheumatisms community support organization in Cyprus, offering the programme as a routine service. All group participants (*n* = 22) were informed about the study and invited to provide feedback; 12 gave informed consent to participate in an interview after reviewing the information sheet.

Eligible participants were adults of all ages, diagnosed with rheumatic disease, including osteoarthritis, rheumatoid arthritis, lupus, scleroderma, fibromyalgia and arthropathy, among others. Exclusion criteria included severe and acute psychiatric comorbidities indicating those with significant functional impairment (e.g., marked psychological disturbance, major cognitive deficits or profound communication difficulties). Recruitment took place between November and January 2021.

The first author (VC), a licensed Counselling Psychologist, with both research and clinical expertise in ACT and an accreditation from the British Association for Behavioural and Cognitive Psychotherapies (BABCP) facilitated the intervention groups. Following the groups' completion, another member of the research team (the second author; KA), who was not involved in intervention delivery and had no prior ACT training, conducted all the post‐programme process evaluation qualitative interviews. This separation ensured that participants could speak candidly about their experiences. Verbal and written consent was obtained prior to each interview.

### Participants and data collection

Ten participants, aged 34–65 years (*M* = 47.25, SD = 9.83), eight of whom identified as female and two as male, all fully attended the intervention. Aggregate demographic details are provided in Table 1 to preserve participant anonymity.

**TABLE 1:** Demographics of study participants.

Demographic information	*N*	%
Age (years)		
Range	34–65	
Mean (SD)	47.25 (9.83)	
Gender		
Female	10	83.3
Male	2	16.7
Employment status		
Employed full‐time	7	66.7
Retired	2	16.7
Unemployed	3	16.7
Marital status		
Married	9	75.0
Single	3	25.0
Economic status		
Low	2	16.7
Moderate	8	66.7
No response	2	16.7
Residence		
Urban	11	91.7
Rural	1	8.3
Number of children		
Mean (SD)	1.83 (1.34)	
None	2	16.7
One	3	25.0
Two	4	33.3
Three	1	8.3
Four	2	16.7
Medical diagnosis		
Spondyloarthropathy	1	8.3
Osteoarthritis	3	25.0
Fibromyalgia	3	25.0
Rheumatoid arthritis	2	16.7
Scleroderma	1	8.3
Psoriatic arthritis	1	8.3
Systemic lupus erythematosus (SLE)	1	8.3

Interviews took place in private, quiet communal spaces and were audio recorded for anonymity, with each assigned an identification number to pseudo‐anonymize content. Interviews averaged 41.44 min and were transcribed manually in full verbatim. All interviews were completed, transcribed and analysed in modern Greek. To ensure translation accuracy, final quotations were directly translated into English. A forward‐only translation procedure was selected, as the data were collected in the Greek Cypriot dialect, which does not usually appear in written form, and for which exact back‐translation was not feasible. Previous qualitative research has noted that back‐translation may be inappropriate in such cases and recommends focusing on direct translations that retain interpretive meaning (Larkin et al., 2007; Ochieng & Nyamongo, 2009). To further ensure fidelity, a consultation process was undertaken between the interviewer (KA) and the first author (VC), both fluent in Greek, English and the Cypriot dialect, to confirm that no meaning was lost (Gawlewicz, 2019). The interviewer completed reflexive notes after each interview to support the analysis (Braun & Clarke, 2019).

### Semi‐structured interview guide

We developed a semi‐structured interview guide (See Table 2) with open‐ended questions addressing acceptability parameters. Following Sekhon et al.'s (2017) acceptability framework, the guide explored five areas related to participants' cognitive and emotional responses to the intervention: (1) motivations and expectations for joining (opportunity costs), (2) reactions to mindfulness concept and practice (affective attitude), (3) personal meaning, values and attitudes towards pain and self (intervention coherence), and (4) application of group skills in daily life (self‐efficacy). In designing the interview schedule, we also considered background literature on the emotional and physical experiences of people living with rheumatic diseases. We considered past literature highlighting the importance of exploring elements such as the multistage process of accepting the disease as crucial to psychological adjustment, the useful role of peer support (Lorig et al., 2009), and the complex psychological impact of the disease, which, when coupled with chronic pain, can result in deteriorating mental health and a tendency to place self and personal needs in a secondary position (Ziarko et al., 2019). The guide included core questions, and where relevant, prompts to facilitate deeper discussion.

**TABLE 2:** Interview guide.

Area of enquiry	Questions
Motivations and expectations from an ACT group	Can you tell me what attracted you to this group? Describe your state of health during the seminar (prompts: explore relationship with body and pain)
	Comment on your emotional state, your mood when the group started (prompts: explore mental health, mood, well‐being) What were your expectations from this group?
Reactions to the concept and practice of acceptance and mindfulness	Can you describe what was your first reaction to the group and to the concepts of mindfulness and acceptance? (prompt: emotional and physical reaction to practices, understanding of practices, newly formed expectations) Can you describe how your reaction to these practices changed over time? (prompt: what was a growing understanding of mindfulness and acceptance, what did they understand mindfulness and acceptance to be at this stage?) Tell me about which practices were most helpful and in what way. Tell me about which practices were most challenging and why. Which aspects of the group instructions were helpful/not helpful to you? (prompt: exercises, metaphors)
Discovering personal meaning and values	During this group there were some exercises and discussions on what is personally important in life. What did you observer or discover through these exercises/discussions? (Prompt: what is important to you? How does mindfulness relate to what is important?) Can you identify and describe the most important moments of the group for you? How were they important to you? Members of the group often talked about the topic of ‘self‐care’, can you discuss this given your personal experience?
Integration of group skills in life	How did you transfer mindfulness into your life after group? What do you think you gained, if anything, through this group? Tell me about how you attempted to practice the group skills in your life (prompt: ways of practice, frequency, barriers, support)
Expressed attitudes towards pain and self	In your experience, how does mindfulness and acceptance relate to physical pain? Can you discuss your relationship with pain before the group? Can you describe some moments during the group or after where you observed a different relationship with pain? (prompt: was anything different?)

### Community‐based ACT group intervention

The group was advertised as a well‐being programme helping participants ‘disentangle from problematic thoughts and sensations, break free from automatic pilot mode, and better navigate unwanted sensations, thoughts, and emotions’. It consisted of five 3‐h sessions, with four held weekly and the fifth following a 2‐week break.

Participants received a booklet summarizing each session and a digital file of mindfulness audio practices. The intervention consisted of elements from the adapted ACT group protocol for the workplace (Christodoulou et al., 2024; Flaxman et al., 2013) and lengthier MBSR practices (Kabat‐Zinn & Hanh, 2013). Each session combined mindfulness practice with the introduction of additional ACT processes. Session 1 introduced the concept of ‘automatic pilot’ and a mindfulness‐of‐the‐body exercise (Christodoulou et al., 2024). Session 2 continued mindfulness and introduced values, including the Bus of Life metaphor and a values clarification exercise (Flaxman et al., 2013; Hayes et al., 2012). Session 3 focused on bodily awareness through mindfulness and incorporated a values‐based 3‐min breathing space (Christodoulou et al., 2024). Session 4 revisited values with the Sweet‐Spot exercise and deepened mindfulness practices (Wilson & Sandoz, 2008). Finally, Session 5 introduced cognitive defusion via the ‘milk, milk’ exercise (Hayes et al., 2012) and included planning for continuing practice post‐group. Adaptations from previous protocols included lengthening ACT mindfulness exercises to support the gradual engagement with bodily discomfort and its connection to personal values. For example, during mindful movement, participants were prompted to reflect on their chosen values. The protocol is included in Table 3.

**TABLE 3:** ACT‐community‐based group guide.

Session number	Interventions
Session1: Automatic pilot	Expectations and introductions to the group Automatic pilot (raisin exercise) (Harris, 2009) Mindfulness of body practice (Christodoulou et al., 2024) Home‐practice: Mindfulness practice, daily mindfulness (e.g., being present during routine activities)
Session 2: Breath and my values	Mindfulness of breath and relationship with thought, emotion, and physical sensation Home‐practice debrief Bus of life metaphor (Hayes et al., 2012) Values definition exercise (Flaxman et al., 2013, p. 224) Home‐practice: Mindfulness of body and breath, mindfulness of daily valued actions
Session 3: The body with difficulty	Mindfulness of breath and sound Home‐practice debrief 3‐min breathing space with values (Christodoulou et al., 2024) Mindful movement connected to valued directions Home‐practice: Any mindfulness practice, mindfulness of daily valued actions
Session 4: Finding the sweetness in life	Mindfulness of breath, sound, and mind Home‐practice debrief Sweet‐spot exercise (Wilson & Sandoz, 2008) Mindful stretch connected to valued directions Home‐practice: Any mindfulness practice, mindfulness of daily valued actions
Session 5: Stepping back from thoughts	Mindfulness of breath, body and mind (clouds in the sky) (O'Donoghue et al., 2018) Home‐practice debrief ‘Milk, Milk’ exercise (Hayes et al., 2012) Thoughts as tools exercise (Stoddard & Afari, 2014) Plan of incorporating group skills into life (mindfulness and values‐based living) Home‐practice: Monthly plan

### Data analysis

We conducted a reflexive thematic analysis (RTA) (Braun & Clarke, 2012) which offers flexibility in choosing theory and epistemology and a structured approach to data analysis (Braun & Clarke, 2022b). Although our approach was primarily inductive, a supplementary deductive lens was adopted, particularly by the first (VC) and third (VSV) authors, using the ACT model to guide thematic interpretation around acceptance, change, personal struggles and meaningful engagement.

Our analyses operated at semantic and latent levels, with an emphasis on uncovering underlying meanings. This flexible method allowed us to stay grounded in participants' voices while identifying central organizing concepts (Braun & Clarke, 2021). To this end, two coders (VC and KA) worked together and iteratively, applying Braun and Clarke's seven‐phase steps for reflective thematic analysis (Braun et al., 2023). The coders were constantly discussing the generated themes and central concepts to reach an agreement on the resultant refined themes. We use illustrative anonymized participants' quotations to validate themes both within the main text and more extensively in the Table S1.

### Quality criteria

We integrated reflexivity throughout the study planning and execution (Braun & Clarke, 2021), acknowledging how the authors' background and beliefs influenced the research process (Creswell & Creswell, 2023). The first author (VC), who facilitated the intervention and led data analysis due to her prior academic experience in qualitative analyses, applied a supplementary ACT‐informed lens to interpret themes around acceptance, change, personal struggles and meaningful engagement. The second author (KA), who was unfamiliar with ACT, led data collection and participated in the analysis without prior theoretical or practical expectations. The third author (VSV), an HCPC‐registered Health and Clinical Psychologist with extensive experience using ACT in individuals with rheumatic conditions, familiarized himself with the data, reviewed the themes and provided feedback on the report. As per the RTA suggestions (Braun et al., 2023), this joint work supported a balanced and transparent interpretive process.

To ensure methodological rigour, we assessed trustworthiness and authenticity, using established criteria (Guba & Lincoln, 1991; Shannon & Hambacher, 2015). Credibility was supported through collaborative analysis, with two coders independently conducting line‐by‐line coding and discussing findings. The third author reviewed the themes, codes and excerpts, offering insights. Authenticity was promoted by identifying negative cases and diverse perspectives (Shannon & Hambacher, 2015). Transferability was supported through rich participant quotes (Guba & Lincoln, 1991). Dependability and confirmability were addressed through an internal audit trail, including memos and reflexive diaries (Nowell et al., 2017).

## RESULTS

### Resulting themes

**FIGURE 1:**
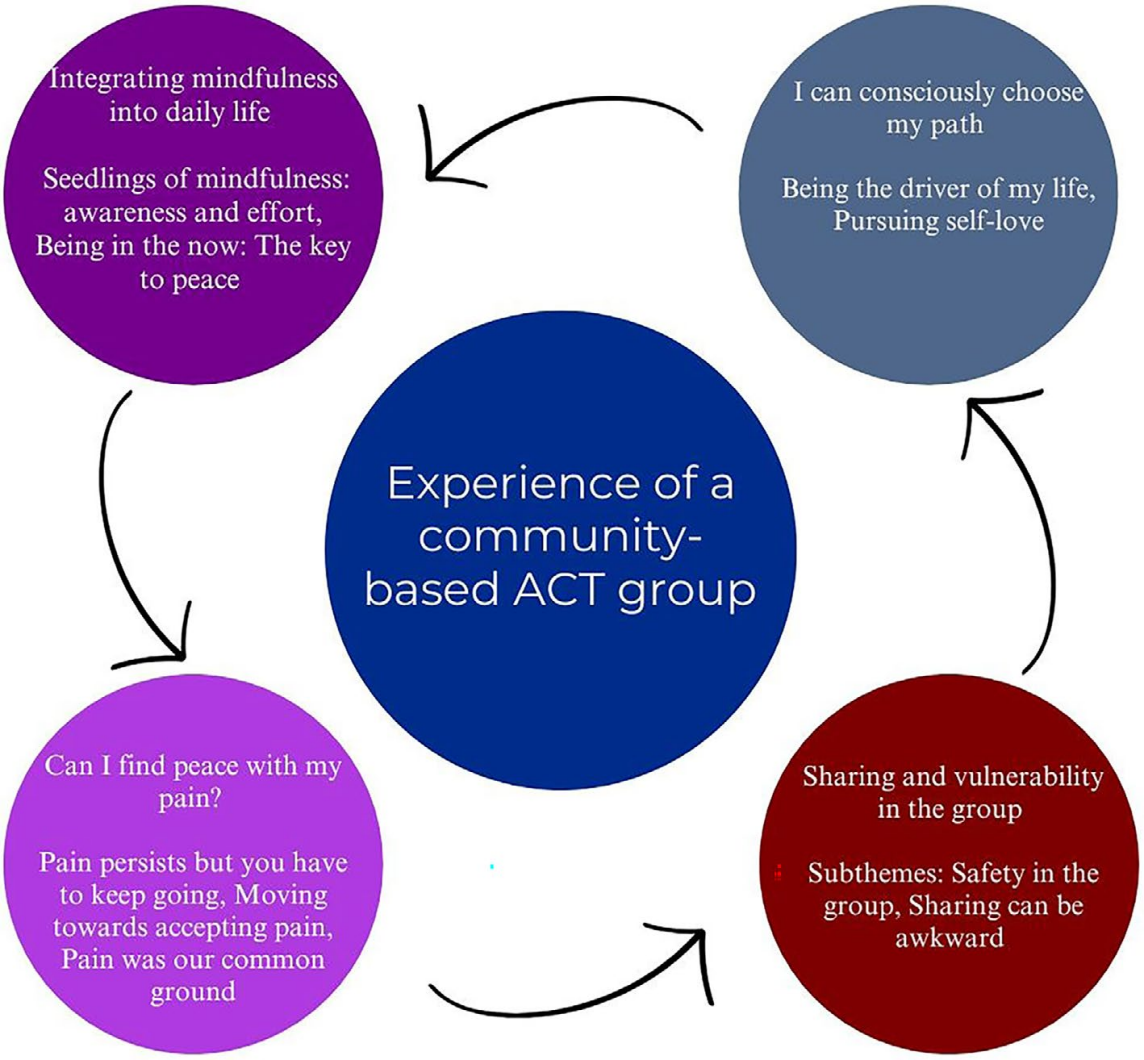
The cycle of ACT community‐based intervention experience.

We identified four themes, each with subthemes capturing participants' group reflections and experiences (Figure 1). The first theme explored how participants integrated mindfulness into their lives and any perceived changes. A second theme summarized values clarification, providing a different approach to life. The third theme focused on participants' conflicting attitudes towards pain. Finally, a fourth theme explored participants' experiences of the group context of the intervention.

### First theme: Integrating mindfulness into daily life

The first theme consisted of two subthemes, reflecting participants' experiences with mindfulness practice and its impact on their emotional and cognitive responses.

#### Seedlings of mindfulness: awareness and effort

This subtheme highlighted how participants introduced mindfulness into their daily routines. Many used the provided audio recordings and informal practices, although this was not a uniform experience. Some struggled to maintain regular practice or found it difficult to return to the present moment. Beyond structured practices, most participants considered experiencing brief, mindful ‘moments’ of increased focus and awareness in everyday activities.

Petros (male, aged 49) said:

What is certain is that this is in my mind every day [mindfulness exercises]. I may not do them. I may catch myself saying, “I shaved, brushed my teeth, I walked into the bathroom without even realizing it.” And the next thing I'm going to do is be more mindful. What does this thing do to me? Those thoughts that arise in the morning, leave the mind […] These things leave my mind, and I rest. I operate with relaxation. … Okay, sometimes I can do my exercises as well.

This passage captures a shared insight into the mindfulness journey, an ongoing movement between distraction and intentional awareness. While participants may be aware of the possibility of practising mindfulness, the automaticity of mundane activities draws them in. Reorientating themselves to the present, even momentarily, indicates a cognitive shift but also a pursuit of a sense of calm.

Stepping out of the routine through mindfulness is also illustrated by Katerina (female, aged 46)

… so… I find this as an opportunity; whereas before I just l… laid down to rest, whereas now I see it as an opportunity. Since I will lay down to rest, I can do something even for a few minutes. One exercise! […]

#### Being in the now: the key to peace

The second subtheme reflected how mindfulness contributed to emotional well‐being by fostering relaxation and an improved quality of life. Participants reported responding to challenges with calmness, a state they had struggled to achieve before.

Magda (female, aged 34) observed:

It helps me accept some things, to… it is what I said… that, many things pass by our lives, our daily life, without us noticing. With some techniques, with some of the things you learn…, new! You can have a different reaction. That is to find, erm… um… calm, peace, relaxation, with things that, in the past you did not even come close, to live the moment.

Many participants also observed a shift in their relationship with thoughts, becoming more aware of the ‘wandering mind’ and developing a more detached perspective.

Panos (male, aged 34) said:

… That I am thinking of some things, I am more relaxed, and we were more relaxed in that moment, and it helped us to see things through a different lens, through a different point of view…

The passages highlight a shift in participants' way of perceiving and reacting to daily life situations, emphasizing an enhanced, more accepting and gentler awareness though mindfulness. This transformative approach, a new lens to see things, appears to provide a more centred and calm perspective and response.

### Second theme: I can consciously choose my path

This theme captured participants' realization of a ‘turning point’ in which they recognized what mattered to them. It reflected a newfound sense of agency—acknowledging that, despite their diagnosis, they still had choices. Two subthemes were identified: *being the driver of my life* and *pursuing self‐love*.

#### Being the driver of my life

Participants described gaining awareness of where they directed their attention, rather than being passively carried away by life's demands. Some acknowledged inconsistencies in terms of their values‐based directions and daily actions. Some participants used the ACT metaphor of the ‘bus of life’ to view themselves as conscious drivers—able to choose what to engage with or acknowledge the difficulty of staying on course while managing ‘challenging passengers’ such as thoughts, pain and feelings.

Magda (female, aged 34) said:

…Erm it's a nice role, when you feel that, when I feel that I am at the steering wheel, because before I felt that life holds, held the steering wheel, in the past tense [emphasis], and takes me where it wants…

The participant highlights an important dimension of living with a rheumatic disease; that is of the lack of control over their physical condition which is characterized by the disease's fluctuating nature. This experience of being vulnerable to the changing nature of the disease can lead to a feeling of helplessness and a lack of agency. However, the participant's reflection represents a process of regaining control and feeling empowered to lead life with intention and awareness. This shift may be reflective of an increased clarity around values and a greater willingness to pursue them, while accepting the unpredictability of physical symptoms.

Katerina (female, aged 46) said:

It was very nice, (pause), very, very, nice, I said ‘I am here with my kids, this moment is mine’, I am living this moment, erm it's priceless, that is what I felt in that moment. (pause). I felt very nice. I felt it! While most times I say, ‘ok my love, come’ but my mind is elsewhere, ‘I will do that chore afterwards or whatever’. […]…. What is important to me are the people who are with me, my family, my friends (pause), to share moments of joy, happiness, (pause) that is important to me. […].

Continuing from an enhanced sense of agency, Katerina's passage shows a shift from passive awareness to engagement in values‐based living. This section illustrates how participants employed present‐moment awareness more deliberately to both acknowledge and connect with meaningful directions connected to their values. This shift towards valuing in daily actions was a key dimension of the programme, as reflected in the introduction of values awareness throughout mindfulness practices (e.g., 3‐min breathing space with values).

#### Pursuing self‐love

Participants described how the programme encouraged them to prioritize and care for their needs. This subtheme highlighted emotional concerns and aspirations, with some participants sharing experiences of neglecting their well‐being due to physical limitations. Narratives in this subtheme revealed a tension between self‐care and responsibilities towards others. Many participants noted becoming gentler and more attentive to themselves.

Katerina (female, aged 46) summarized this:

What I really liked, and it fits with my temperament, and it fits with the whole concept of mindfulness is that there is nothing that you ‘must’, let it be and look at yourself with compassion and understanding.

Magda (female, aged 34) also said:

… It is important for me to be able to offer to those around me, to my family. This makes me happy. But I also want to be okay with myself. That is, I want to also have my own time, and my own space to improve myself.

Individuals living with a rheumatic disease often manage limited physical resources, and in the context of many obligations, self‐directed kindness is often not a priority. The passages highlight a struggle to find the gentle balance among these responsibilities, while moving away from internalized, forced rules that may feel both demanding and oppressing. This stance reflects the gentleness embedded in the programme, where ‘approaching the difficult’ is offered as an invitation rather than a requirement.

### Third theme: Can I find peace with my pain?

The third theme represented the internal debate between accepting and controlling pain and symptoms. Despite varying levels and types of physical complaints, participants shared narratives of an ongoing negotiation between tolerating pain and symptoms (viewing it as something unavoidable) and avoiding it (as something to be rejected or suppressed). This included two subthemes, capturing efforts to maintain a balancing stance and an interplay battle between acknowledging and controlling the symptoms.

#### Pain persists but you have to keep going

The first subtheme emphasized the omnipresence of pain, physical discomfort, and its emotional toll and impact on daily interactions and activities. Participants presented with a dynamic relationship with discomfort, recognizing that the programme would not alleviate their pain. However, their distress, which remains recurrent and unpredictable, can potentially intensify their sense of vulnerability; hence, it represents a more realistic goal.

As Athina (female, aged 46) noted:

…lately the joint pain has returned, erm and ok. This is what I am going through this period, and I cannot say that it helped at all or whether they help at all these…, these…., these methods.

Participants also expressed an emotional response to their physical discomfort, often feeling frustration or sadness, which sometimes conflicted with their desire to make meaningful life choices. This state often triggered a perceived sense of being different to others, often accompanied by bitterness or a feeling of being misunderstood (even by the facilitator). This aligns well with literature on individuals with rheumatic diseases who face interpersonal challenges, as the fluctuating nature of symptoms may lead others to perceive them as lazy or lacking motivation (Lacaille et al., 2007).

Illustratively, Georgia (female, aged 65) said:

Erm what I told you about the trainer, when I told her, erm I felt, I felt that she, that ‘you don't get me now’. Erm the example doesn't get us, let's say that the example cannot be applied to us [refers to the bus metaphor]. What are you telling us now? I mean we want to do things; we want to solve it and the pain won't let us live the life we wanted to live. Erm… to do the normal things everybody else does, for us, it is a big deal.

Some participants described efforts to maintain a controlled distance from their pain reflecting psychological processes consistent with experiential avoidance, as described in the psychological flexibility model, presenting the six facets that lead to suffering, one being experiential avoidance (McCracken, 2024).

Niki (female, aged 53) shared a vivid mental image of how she managed her pain to contain it:

Yes, I can imagine a ball, let's say that it's the problem, it looks like filled with thorns and wherever it touches it strikes, and it burns. Erm, in this way, let's say, that you can put your foot down, I will not let this thing, this evil get inside me and… and…

Participants also mentioned the burden of continuing daily activities while in pain, illustrating the constant and emotionally taxing negotiation with pain under a relentless demand of daily responsibilities.

Vasiliki (female, aged 36) said:

The truth is that even if I am in pain many times, many times, I have to reach my limit. I must do this for the children, that for my husband, this for work, that for my friend, this…

#### Moving towards accepting pain

The second subtheme indicated the non‐linear, tentative process of practising pain acceptance where participants momentarily create space for physical discomfort without allowing it to dominate, through noticing and allowing it to be present. This subtheme therefore reflects an experience closely aligned to the ACT process of acceptance, which has been found to follow a gradual trajectory of growth in individuals living with chronic pain (Scott et al., 2016). In most narratives, this practice was fleeting and difficult to maintain, yet participants acknowledged its potential emotional benefits.

Katerina (female, aged 46) highlighted the impermanence of this experience:

Most of the time I was drowning in the sea, but the few, the very few times that I realised that [pause] erm, [pause] the pain is there but I do not need to suffer because it is there, I felt better [pause]. But this happened very few times [audible: sad smile].

On the other hand, Gianna (female, aged 48) described a more allowing and compassionate stance towards pain, more reflective of a gradual shift away from a forceful attempt at control, and towards observing, making space, and reengaging. This attitude is more consistent with the gentle, non‐judgemental awareness cultivated in ACT.

Before, […] I would do something and feel pain in my arm and I would say, let's say, ‘but why, for example, should I be in pain and not be able to keep going?’ Whereas now, let's say, I might say, ‘okay, I'll stop, do something else, let's say, rest and then continue.’

### Fourth theme: sharing and vulnerability in the group

In this fourth theme, participants reflected on their group intervention experience and the sense of connection they felt with similar others. The theme is organized into three subthemes incorporating perceived similarities among participants as well as challenges in the group context.

#### Pain was our common ground

Participants disregarded differences such as age, gender or other characteristics and focused on how the experience of living with a chronic illness connected them. While pain was generally viewed negatively, it fostered a sense of camaraderie that helped build relationships. Pain, in this subtheme, was not only a symptom but a thread connecting participants that created a sense of belonging. This subtheme may represent a contrast to participants' earlier experiences of feeling different and misunderstood by others (presumably those not living with a rheumatic disease) as identified in the third theme: Can I find peace with my pain?

Niki's narrative (female, aged 53) illustrates a sense of relief of being a member of a group where pain was not a point of debate or judgement but a common, well validated experience. She said:

Yes… The pain. The pain and when… the first time you meet a person, because before you meet… erm many times you are afraid to express yourself and… you are perceived as dramatic… erm but when you turn to someone who suffers, and it is not something that has an expiration date, you share with them some things and then…, and I think on the topic of pain… well more or less we all expressed ourselves at certain parts.

#### Safety in the group

In the second subtheme, most participants described feeling safe and open with the group. They found sharing their experiences interesting and pleasant. Some developed friendships beyond the group.

Rodanthe (female, aged 51) said:

It felt very familiar. The environment was generally very pleasant. I did not feel any pressure or negative feeling, or to… I felt very comfortable and to express myself and easy. That is, I waited for the meeting each time.

The anticipation for attending the group, illustrated in this narrative, reflects the creation of a secure space in which one felt free to simply ‘be’. This experience probably led to a sense of relief in contrast to the pressures of the daily life.

#### Sharing can be awkward

The experience of the group was not uniformly easy, as some participants found sharing emotions challenging. Group dynamics were slow to develop, and it took time to feel comfortable opening up emotionally.

Vasiliki (female, aged 36) said:

Therefore, I felt a familiarity and that I can also do it more easily maybe because I am a bit shy; Usually I only accept things in a passive way. Here I felt that I could express myself. Of course, not much as I… I feel that I would [share] more.

Emotional expression was portrayed as a gradual process in this subtheme, and for some participants, this may have echoed a previous tendency to hide one's inner experience. Their gradual willingness to acknowledge and share may be reflecting the growth of emotional safety in the group or even the slow acceptance of their own inner experience.

## DISCUSSION

Community‐based programmes offer a promising approach for delivering psychosocial interventions to individuals with rheumatic conditions. However, little is known about how such interventions are perceived in community contexts. Unlike prior studies that primarily focused on examining effectiveness outcomes (e.g., Hegarty et al., 2020), in this study, we implemented a qualitative process evaluation to explore how participants engaged with and interpreted ACT processes, such as mindfulness, values and acceptance, in managing their symptoms. We used the theoretical framework of acceptability (TFA; Sekhon et al., 2017) to explore key dimensions of acceptability—including affective attitudes, perceived effectiveness, and intervention coherence—within a community ACT programme.

Existing research has demonstrated that ACT can enhance psychological flexibility (Bendelin et al., 2020; Köhle et al., 2017), promote values‐based living (Rise et al., 2015) and reframe symptom experiences (Keenan et al., 2024; Thompson et al., 2018). Our findings build on this by highlighting the acceptability and feasibility of ACT within a community‐based framework, shedding light on how participants perceived its mechanisms of change. Our study aligned with the pragmatic Contextual Behavioural Science recommendation 32 (Hayes et al., 2021) calling for research that expands access to mental health resources by deploying interventions in non‐conventional clinical settings. Conducting naturalistic research is essential, given the common availability of psychosocial support through local organizations and charities. This aligns with our process evaluation approach, exploring how interventions are replicated in context by examining change processes and contextual factors (Moore et al., 2015). In short, our findings indicate that a brief ACT‐based group intervention was well accepted and that participants engaged with values‐based actions, committed efforts, and mindfulness—focal ACT processes that shaped their experience of the programme.

Our findings align with previous quantitative ACT studies, demonstrating beneficial outcomes for managing chronic conditions (Graham et al., 2016; McCracken & Vowles, 2014). Additionally, in line with previous ACT qualitative studies with other chronic conditions, the intervention fostered greater attentiveness to personal needs (Keenan et al., 2024), encouraged resource allocations towards meaningful actions (Rise et al., 2015; Thompson et al., 2018), and, albeit reluctantly, supported some symptom acceptance (Bendelin et al., 2020; Thompson et al., 2018).

Yet, our intervention identified some important disease‐specific parameters that underscore the nuances and intricacies of rheumatic conditions. These findings can inform the development of tailored psychosocial care programmes addressing the specific needs of individuals with rheumatic conditions. Below, we discuss our findings in relation to existing literature.

Firstly, three of our identified themes, namely, “Mindfulness in my life”, “I can consciously choose my path”, “can I find peace with my pain?” highlighted participants' experiences with cultivating mindfulness. While they recognized its potential benefits and reported a stronger connection to the present moment, our findings challenge the expectation that mindfulness can be easily consolidated as a lifelong skill. A review of qualitative studies of mindfulness interventions for cancer survivors highlighted similar themes, particularly the tension between cultivating present‐moment awareness and the difficulty of fitting mindfulness practices into their daily lives (Tate et al., 2018). However, this process may not be simple. Other studies with chronic health populations found that mindfulness could sometimes trigger avoidance, as participants experienced the mindfulness practices as unwanted reminders of illness (Eyles et al., 2015; Hoffman et al., 2012; Tate et al., 2018)—a challenge not reflected in our participants' accounts. As we comment below, we believe that the reported difficulties with mindfulness may stem from two key factors: time constraints in formally practising mindfulness and deeply ingrained attitudes and beliefs about pain (Lööf, 2019).

For the barrier with time constraints, shorter daily mindfulness practices, tailored to physical limitations (Glynn et al., 2020), may be more sustainable for individuals with rheumatisms. In fact, participants in our study provided examples of spontaneous use of mindful awareness during everyday activities (e.g., brushing teeth, resting). Regarding the second barrier referring to pain‐related attitudes, individuals with rheumatic conditions often experience heightened body awareness, which can trigger automatic interoceptive judgements (Costa et al., 2019) and symptom catastrophizing (Costa et al., 2014). As a result, practising non‐reactive mindfulness presents a unique challenge because the default responses to pain are driven by automatic interoceptive body biases (Biguet et al., 2016; Costa et al., 2019). A key adjustment may be helping individuals shift from seeing their pain as a threat to simply observing it with mindfulness. This approach can also encourage a more balanced view of pain, such as recognizing when to rest and when to engage in meaningful activities. Supporting this shift may be essential for successfully integrating mindfulness into rheumatism care. However, this process may not be simple and may require the recognition of individuals' barriers. Other qualitative studies have identified avoidance of mindfulness practices, with participants reporting that practising was an unwanted reminder of their illness (Eyles et al., 2015; Hoffman et al., 2012; Tate et al., 2018)—a challenge not reflected in our participants' narratives.

Secondly, our findings identified the role of values and committed action as two key ACT processes of change for this population. The theme ‘I can choose my path' captured participants’ reflections on the role of values‐based living, or the lack of values‐based living, and the realization that actions can be guided by personal priorities rather than the progression of their condition or external obligations. Participants described how the programme helped them renegotiate their values—reassessing commitments, balancing expectations (e.g., pre‐diagnosis assumptions about physical activity levels) and aligning them with the realities of their condition in a more contextually appropriate way. In fact, participant narratives highlighted mindful awareness as a facilitative process in recognizing personally meaningful actions and values (i.e., ‘…While most times I say, “ok my love, come” but my mind is elsewhere…’). Although not always explicit in our data, individuals with rheumatic disease may at times de‐prioritize values‐based actions, including self‐care, in balancing illness demands with everyday responsibilities. This pattern echoes findings in prior qualitative work with this population (Kristiansen et al., 2012a, 2012b) and aligns with studies of ACT‐based rehabilitation in other contexts. For example, Rise et al. (2015) described participants shifting towards values clarification as a way to reconcile external pressures with personal meaning, a process reported as both liberating and empowering (e.g., ‘… more grateful to the universe for what I have and realise what really matters in our life’.)—similar to experiences documented in ACT studies with cancer populations (Balta et al., 2025; Keenan et al., 2024). The findings underscore the role of psychological flexibility in helping individuals with chronic physical conditions reconsider what is personally meaningful, enabling a balance between external demands, societal expectations, and the need for self‐care in the face of illness‐related limitations (Kristiansen et al., 2012a, 2012b).

Thirdly, the theme ‘Can I find peace with my pain?’ highlighted the complex and dynamic role of pain acceptance in rheumatic conditions. Participants acknowledged the need to stay engaged in activities but approached pain with tolerance. Their narratives often revealed a sense of frustration and anger towards pain and others' expectations, reflecting an internal struggle between accepting and attempting to control it. Notably, an allowing stance towards pain was rare, aligning with previous research suggesting that individuals with rheumatic conditions often struggle with a willingness to experience pain (Costa et al., 2019).

This dynamic interplay between ‘accept and control’ is influenced by the fluctuating nature of rheumatic conditions, which are marked by periods of symptom remission and flare‐ups (Javed et al., 2022). Unlike individuals with persistent chronic pain, those with rheumatism do not experience pain continuously, but during the periods of inflammation, which may shape their approach to acceptance differently. As evident in our narratives, this fluctuation may contribute to feelings of helplessness and vulnerability, along with a sense of being misunderstood or judged by others for not meeting expectations—an observation also noted by Lacaille et al. (2007). Other qualitative studies have arrived at similar themes. For instance, Bendelin et al. (2020) identified a ‘control or command’ theme in participants undergoing internet‐based ACT for chronic rheumatic pain, where willingness to engage with pain was framed as a strategy to exert control over it. Moreover, other qualitative studies also identify barriers to acceptance, akin to avoidance (Eyles et al., 2015; Hoffman et al., 2012; Keenan et al., 2024; Tate et al., 2018).

Findings from the third theme underscore the need to focus more on cognitive processes that may influence how individuals approach pain acceptance. Quantitative studies have consistently shown that fear‐avoidance beliefs (Lööf et al., 2015; Lööf & Johansson, 2019) shape how people with rheumatic conditions interpret and respond to pain (Lööf, 2019). For example, cognitive constructs, such as reappraisal (De Vincenzo et al., 2024), self‐efficacy (Ahlstrand et al., 2017) and catastrophizing (Shim et al., 2018; Tighe et al., 2020) have been identified as independent mediators in the relationship between pain and well‐being, either facilitating or interfering with pain acceptance. Emerging neuroimaging studies also suggest that pain acceptance can modulate problematic pain perceptions by influencing brain network activity, particularly with fluctuating symptoms like chronic pain (Vasiliou et al., 2025). A final, speculative, yet important consideration is the potential cultural influence on pain acceptance. Greek‐speaking individuals—the population in this study—have been shown to exhibit high levels of experiential avoidance compared to other European cultures (Monestès et al., 2018), a pattern also observed in other studies with individuals with chronic pain (Vasiliou et al., 2019; Zacharia et al., 2021). These cultural tendencies may contribute to resistance towards pain acceptance practices. Taken together, these findings underscore the need for more research into the cognitive and emotional mechanisms that shape pain experiences in rheumatic conditions, particularly across diverse cultural contexts.

The final theme reflected a well‐replicated finding: the positive impact of belonging to a group with shared experiences (Chambers et al., 2012). Despite its consistency, our findings reinforce how shared concerns can normalize condition‐related experiences. However, in line with our effort to fully analyse the data, looking for negative cases (Fife & Gossner, 2024), some participants reported discomfort and difficulty fully expressing themselves. While less common, this aligns with digital self‐help intervention studies that show the value of anonymity, the low need from some individuals to receive peer support, and individuals' fears of confronting others' stories (Köhle et al., 2017). Similar findings were also reported in a qualitative ACT study with stroke survivors where some participants reported feeling discomfort in the presence of unfamiliar people and others experiencing openness and trust (Storey et al., 2025). Given the scarcity, yet presence of this finding in our study, future research should explore the perspectives of individuals from online versus face‐to‐face interventions. Some participants may likely prefer online ACT applications for accessibility and convenience, while others may engage in experiential avoidance through this format. In both cases, elements of personalization in intervention might increase engagement and expansion of evidence‐based psychosocial programmes for this population.

### Limitations

Certain limitations must be considered. Firstly, the female gender was overrepresented both in group and study participants, indicating that the group may not be as attractive to other genders. Secondly, in accordance with informed consent procedures, not all group participants agreed to participate in the interview study. Although their disease profiles were similar to those who did participate, it remains unclear whether non‐consent was due to dissatisfaction or for other reasons. Moreover, the findings may reflect only a subgroup of individuals living with a rheumatic diagnosis, as recruitment was based on voluntary participation—a method that is likely to attract individuals who are more motivated and available to engage. Further, despite the range of ages and professional statuses among participants, we did not collect information on their educational level, limiting our understanding of whether the sample reflected a particular educational profile.

Although participant comments were generally positive towards the intervention, the risk remains that participants may have felt uncomfortable expressing dissatisfaction, even though the person delivering the group (VC) was different from the interviewer (KA). However, interview narratives were not excessively positive or uncritical. Participants openly discussed challenges with mindfulness practice and expressed ambivalence towards pain. This nuanced feedback increases our confidence in the validity and trustworthiness of the findings.

A final key limitation of this study was the absence of formal fidelity assessment, as video or audio recordings of group sessions were not feasible in this naturalistic community setting. Future research should consider embedding recording methods to enable within and post‐intervention fidelity checks, for example using the ACT‐FM grid (O'Neill et al., 2019). Nonetheless, several factors support confidence that our community‐based ACT programme was delivered as intended: the facilitator and advisor both had strong ACT expertise, a structured and protocolized manual was used consistently, and participant testimonials directly referenced ACT processes, including mapped data (quotes) showing processes of values, mindfulness and acceptance.

### Implications for practice and policy

The findings of this study have several implications. First, they support the acceptability of delivering Acceptance and Commitment Therapy (ACT) in a brief format for individuals living with rheumatic diseases, while preserving a relatively accurate interpretation of the ACT model. This aligns with the priorities outlined in the Association for Contextual Behavioral Science (ACBS) Task Force report, particularly the recommendation that ‘CBS research needs to help ensure that research that meets human needs is promulgated and used’ (Hayes et al., 2021, p. 182).

Secondly, a key implication is that a brief programme may not be sufficient to support a transformative relationship with disease symptoms, particularly pain. This was evident in participants' ambivalent narratives regarding their pain experience, and other research indicates similar findings (Taylor et al., 2016). Our results suggest a dynamic interplay between accepting physical pain and acknowledging limitations. Both outcomes are being driven by rheumatic conditions, which present with symptoms' fluctuations and unpredictability. Ultimately, this challenges the linear progression of therapeutic change. Likewise, mindfulness was sometimes described by participants as a strategy for controlling symptoms. This highlights the complex and non‐linear nature of cultivating mindfulness in the context of chronic conditions. In this context, we suggest that community‐based group programmes for this population should include elements of personalisation and tailoring ‘bites’ of the intervention to participant needs. For instance, supporting organizations may offer screening programmes to identify participants' needs before group participation, followed by a hybrid group and individualized or coaching sessions tailored to specific challenges. The inclusion of monthly follow‐up booster sessions may be essential for reinforcing group cohesion and sustaining therapeutic gains. Given the nuanced findings around mindfulness, a stepwise delivery approach may be beneficial. This could involve preparatory components delivered sequentially, focused on body awareness, interpretation of symptoms, and the gradual integration of mindfulness into daily life. In the current study, daily mindfulness practice was linked to values‐based actions. According to participant narratives, this structure facilitated the continued application of mindfulness and values‐based living after concluding the group sessions.

## CONCLUSIONS

The value of conducting process evaluation qualitative research within ACT‐based interventions lies in uncovering participants' interpretations of the community delivery and the underlying processes of change. Our evaluation provided insight into participants' lived experiences as the intervention unfolded and helped identify individual needs that can optimize the delivery of ACT‐based community programmes.

The findings highlight that participants engaged with and understood core ACT processes, shedding light on the nuanced ways in which certain processes of change, or ‘kernels’—such as mindfulness and acceptance—can be effectively delivered to optimize impact for individuals with rheumatic conditions. The concept of values was found to unlock committed directions that had previously been neglected or unpursued. We conclude that a community‐based intervention, delivered by a supporting organization, may serve as a feasible and acceptable step‐care psychosocial service for this population. Additionally, our findings underscore the need for further research into the evolving nature of mindfulness practices and the acceptance of pain. Future studies should consider longitudinal and idiographic research designs to more effectively capture the complex and evolving trajectories of mindfulness and pain acceptance over time and within individuals.

## AUTHOR CONTRIBUTIONS

**Vasiliki Christodoulou:** Data curation; formal analysis; writing – original draft; writing – review and editing; project administration. **Katerina Artemiou:** Data curation; investigation; project administration. **Vasilis S. Vasiliou:** Conceptualization; methodology; supervision; writing – review and editing.

## CONFLICT OF INTEREST STATEMENT

Vasilis Vasiliou holds editorial roles at the Journal of Contextual and Behavioural Science (JCB) and the British Journal of Health Psychology (BJHP) and reports a relationship with Wanax Health Care Solutions Ltd. that includes ad hoc Health Psychology consulting or advisory roles.

## Supporting information


Table S1.

## Data Availability

The data that support the findings of this study are available from the corresponding author upon reasonable request.
