# Supplementary material for: Community‐based Acceptance and Commitment Therapy Programmes for rheumatic conditions: An acceptability and qualitative process evaluation study
Source: Br J Health Psychol. 2025 Oct 23;30(4):e70031. doi: 10.1111/bjhp.70031 (PMC12548317; doi:10.1111/bjhp.70031)
Supplement: Supplementary file 1 — Table S1. [file BJHP-30-0-s001.docx]

# S1. Thematic Table

| Theme | Subtheme | Example code labels | Example excerpt |
| --- | --- | --- | --- |
| Integrating mindfulness into daily life | **Seedlings of mindfulness: awareness and effort** | Stepping out of autopilot | “I try to see… the sky…, to see the clouds a bit, to make shapes; the other that… I try to see, a bit, in the car, the environment around me, I try” (1) |
|  | **Growing awareness** | Connect with the present moment | "Now I believe that I am probably at an infant, fetal level. Buut… erm, I realise thaat… if someone manages it, to the extent, to the extent that one manages to live in the present moment… erm it’s a key to happiness." (4) |
|  |  | Mindful of everyday activity | "I wake up in the morning feeling pressure to get to work in a hurry. And I start the routine to do everything quickly. To go to the bathroom, to shave etc. To the point that sometimes I say to myself "but when did I shave?" I don't remember when i shaved. When i see that i am in pressure, i stop and i do everying mindfully. I mean even openning the shaving cream, to apply the cream on my face, to look at mysefl, to get the razor." (11) |
|  | **Effortful, yet new** | Time is a barrier to practice | "It's what we say that "I don't have time now to lose twenty minutes to do the exercice"! It's happened to me the last few days…Let's say that during that time that I was going to go, it would have been a nice time to do the exercise, but then I say "I am in a hurry, i have to go". |
|  |  | Mind drifting | "Well my mind started running interference… "why, and how, and what's gonna happen and whatever, and this and that", by the time you realise… ermm they dissapear (the thoughts), "follow the instructions and we will see…". (4) |
|  |  | Practical limitations to practice | “It’s more about, let’s say erm my kids are home, let’s say... and someone will walk in and someone will walk out. I also have a dog that can bark…" (6) |
|  |  | Attempting to practice | "The most important thing is that I want to practice it even more. I mean, I want it to become a way of life. Whereas now I am in the process that I have to think about it to do it" (8) |
|  |  | It’s something new | "Positive, positive, it was something different. We had touched on something similar in (name of another program) with some exercises, let's say, with some relaxation techniques, buut…" (5) |
|  | **Being in the now: The key to peace** | A different way of looking at things | "I would have said (to a friend) that you will be surprised ow nice you feel feel when (…) you start to apply the short or the long exercises they will give you, and you will see things from a perspective that you never saw them before" (4) |
|  |  | Accepting | "Slowly, slowly I said that this way will not help me. I have to accept, I have to accept the fact that, my pain…, I have to accept the siutaiton that I am in, so it helped me on this topic, to accept the things as they are." (1) |
|  |  | Improved quality of life | "Better quality of life, I think. It gives you quality from the moment that you start to enjoy the now." (8) |
|  |  | Stress management | "…I used to get anxious causing…, not too much but I did get anxious. Erm… the seminars, with their help, somehow I realised that I do not need to get so very anxious". (7) |
|  |  | Relaxation | "…Coming home and feeling more restful, waking up to go to work feeling relaxed, joyful!" (11) |
|  |  |  |  |
| I can consciously choose my path | **Being the driver of my life** | Attending to what matters | "…when I am with my parents or with other people erm… but especially with my parents because they are older (…) I prepare myself and I say 'now that I will see them I will give them all my attention of my being there, not to get distracted like I used to..." (1) |
|  |  | My choice | "To try to recognise… to do, let's say, those things that you want and to not think too much about the rest of the world…" (2) |
|  |  | The 'bus' metaphor is helpful | "The story, erm, I liked it a lot. I mean erm I told it to others and we thought about it, talked about it, erm I think that that story helps a lot." (12) |
|  |  | Staying consistent with values is difficult | "Before I gave more dedication to my work. This, I don't know if it's good. Not that I don't know, I know, but I don't feel it! And… I want to send some time for (name) and for the people that I love." (7) |
|  | **Pursuing self-love** |  | "While this now, ok, I have not stopped loving my children nor the people around me, but that behaviour where I would sacrifice myself, I do not do that anymore" (5) |
|  |  |  |  |
| Can I find peace with my pain? | **Pain persists but you have to keep going** | Activity with pain | "Ok, when you are in a lot of pain and you will lay down to sleep... you will do various things. But you still have to do duties etc. So there is no chance that you will not feel the pain, not to be troubled by it. But while I am with other people I have to be in a different state." (9) |
|  | **Moving towards accepting pain** | Hard to accept pain | "…many times I may have felt that the pain affected me, like I had not accepted it. I had anger, a lot of anger (…). It didn't change (relationship with pain). It is trying to change. Yes, I think that it is friendlier but it has a long way to go still. I think it still needs a lot of work from me." (8) |
|  |  |  |  |
| Sharing and vulnerability in the group | **Pain was our common ground** | We had similar problems | "Ok, this was good that (?) that, there are others that have the same problem or a simlar problem to yours." (3) |
|  |  | Pain united us | "You are in a place in that moment that you share, you have a commonality, the pain…" (5) |
|  | **Safety in the group** | Felt safe |  |
|  | **Sharing can be awkward** | Uncomfortable to share | "You don't express yourself easily like that, to… share somethings because, you don't know how the other members of the group might react" (5) |
|  |  | Different experiences | "When I heard the feedback afterwards, that 'I felt this', because I want not interesting at that time, I was doing it more cognitiively than… So I said, ok we are talking in a different dimension. If I was with people who were there to manage their anxiety, like i was, we were definately going to be talking about other things" (10) |
